# Trends in Lung Cancer Incidence Rates by Histological Type in 1975–2008: A Population-Based Study in Osaka, Japan

**DOI:** 10.2188/jea.JE20150257

**Published:** 2016-11-05

**Authors:** Fukuaki Lee Kinoshita, Yuri Ito, Tomio Nakayama

**Affiliations:** 1 School of Medicine, Osaka University, Suita, Osaka, Japan; 1 大阪大学医学部医学科; 2 Center for Cancer Control and Statistics, Osaka Medical Center for Cancer and Cardiovascular Diseases, Osaka, Japan; 2 大阪府立成人病センターがん予防情報センター

**Keywords:** cancer, lung cancer, incidence, histological type, cancer registries, がん, 肺がん, 罹患率, 組織型, がん登録

## Abstract

**Background:**

Monitoring trends in lung cancer incidence and mortality is important for the evaluation of cancer control activities. We investigated recent trends in age-standardized incidence rates by histological type of lung cancer in Osaka, Japan.

**Methods:**

Cancer incidence data for 1975–2008 were obtained from the Osaka Cancer Registry. Lung cancer mortality data with population data in Osaka during 1975–2012 were obtained from vital statistics. We examined trends in age-standardized incidence and mortality rates for all histological types and age-standardized incidence rates by histological type and age group using a joinpoint regression model.

**Results:**

The age-standardized incidence rate of lung cancer levelled off or slightly increased from 1975–2008, with an annual percentage change of 0.3% (95% confidence interval [CI], 0.1%–0.4%) for males and 1.1% (95% CI, 0.9%–1.3%) for females, and the mortality rate decreased by 0.9% (95% CI, 1.2%–0.7%) for males and 0.5% (95% CI, 0.8%–0.3%) for females. The incidence rates of squamous cell carcinoma (SQC) and small cell carcinoma (SMC) significantly decreased for both genders, whereas that of adenocarcinoma (ADC) significantly increased among almost all age groups in both genders.

**Conclusions:**

The incidence rates of SQC and SMC decreased with the decline in smoking prevalence, which probably explains the change in trends in the incidence rates of lung cancer from the mid-1980s. However, the reason for the increase in ADC remains unclear. Therefore, trends in incidence rates of lung cancer should be carefully monitored, especially for ADC, and the associations between ADC and its possible risk factors should be studied.

## Abstract (ja)

**背景：:**

肺がんの罹患率や死亡率の傾向を調査することは現状のがん対策を評価していく上で重要である。本研究では、大阪府における肺がんの組織型別罹患率の推移を調査した。

**方法：:**

肺がんの罹患については大阪府がん登録より1975～2008年診断分のデータを入手した。肺がんの死亡については人口動態統計より1975～2012年死亡分のデータを入手した。joinpoint regression model を用いて、肺がん全体の年齢調整罹患率と年齢調整死亡率、また、組織型別、年齢区分別の年齢調整罹患率の推移を解析した。

**結果：:**

肺がんの年齢調整罹患率は男性においては年平均変化率0.3% (95%信頼区間: 0.1～0.4%)で横ばい、もしくは、わずかに増加していたが、女性においては1.1% (95%信頼区間: 0.9～1.3%)で増加していた。年齢調整死亡率は男性においては－0.9%で、女性においては－0.5%で減少していた。組織型別の解析では、扁平上皮癌と小細胞癌は男女ともに有意に減少していたが、腺癌は男女ともにほとんどすべての年齢区分で有意に増加していた。

**結論：:**

扁平上皮癌と小細胞癌の罹患率は喫煙率の減少とともに減少しており、それがおそらく、1980年代半ば以降における肺がん全体の罹患率の傾向の変化と関係していると考えられる。しかし、腺癌が増加している原因はよくわかっていない。したがって、これからも肺がんの罹患率の傾向、特に腺癌の傾向を調査し、腺癌とその危険因子との関係を明らかにしていく必要がある。

## INTRODUCTION

In Japan, incidence rates of lung cancer have levelled off for males and are increasing for females. Mortality rates of lung cancer show a decreasing trend for males and have levelled off for females.^1^ It was previously reported that incidence rates of lung cancer in Osaka had levelled off for males but increased for females, and that mortality rates showed a slightly decreasing trend for males and females in an analysis using the joinpoint regression model.^2^

Smoking is a major risk factor for lung cancer. The population attributable fraction of active smoking to lung cancer mortality is about 70% for males and 20–40% for females.^3^^,^^4^ However, trends for lung cancer incidence vary by histological type. Previously, it was reported that incidence rates of adenocarcinoma (ADC) increased and incidence rates of squamous cell carcinoma (SQC) and small cell carcinoma (SMC) decreased for both genders in Osaka, Japan.^5^^,^^6^ Incidence rates of SQC and SMC increased among younger groups in their 40s and 50s and older groups aged more than 70 years in the 1990s, but decreased or levelled off among intermediate groups in their 60s. Incidence rates of ADC were reported to increase among most age groups.^7^

In the present study, we updated the trends in lung cancer incidence and mortality rates for all histological types and estimated incidence rates by histological type and age group in Osaka, Japan.

## METHODS

Lung cancer incidence data for 1975–2008 were obtained from the Osaka Cancer Registry (OCR). Lung cancer mortality data in Osaka from 1975–2012 were obtained from vital statistics. Population data by sex and 5-year age group in Osaka were obtained from the National Census. This study was approved by the data usage committee of the OCR at the Osaka Medical Center for Cancer and Cardiovascular Diseases (Osaka, Japan) in August 2014 (approval ID: No. 14-0008).

When analyzing incidence rates by histological type, we followed the histological classification for lung tumors published by the World Health Organization.^8^ Histological types were categorized as follows: SQC (International Classification of Diseases for Oncology Third Edition, Morphology [ICD-O-3M]: 8050–8078, and 8083–8084), ADC (ICD-O-3M: 8140, 8211, 8230–8231, 8250–8260, 8323, 8480–8490, 8550–8551, 8570–8574, and 8576), SMC (ICD-O-3M: 8041–8045, and 8246), unspecified malignant neoplasm (ICD-O-3M: 8000–8005), and other specified malignant neoplasm. The data from the OCR included cases without specific histological diagnosis and stage. To include the missing data for histological type and stage in our analysis, we applied multiple imputation (MI).^9^^,^^10^ For the imputation, we used a multinomial logistic regression model that included another incomplete variable and the complete variables: sex, age at diagnosis, period of diagnosis, and vital status. For the MI method, we used the *ice* command in Stata version 12 (STATA Corporation, College Station, TX, USA) and obtained 10 complete data sets.^11^^,^^12^ When analyzing incidence rates by age group, age at diagnosis was classified into three categories: 35–64 years old, 65–74 years old, and over 75 years old, which were age-standardized within those age ranges.

First, we calculated annual age-standardized incidence and mortality rates (ASR) of lung cancer for all histological types and truncated age-standardized incidence rates by age group. We used the Japanese model population for 1985 to standardize age distribution. When analyzing by histological type, we used the 10 complete data sets obtained from the MI method. Second, we applied the joinpoint regression model^13^^,^^14^ to identify the years when the statistically significant changes in incidence or mortality trends occurred using the Joinpoint Regression Program 4.1.0 (National Cancer Institute Surveillance Research Program Statistical Methodology and Applications Branch, Bethesda, MD, USA).^15^ In the joinpoint analysis, we used the logarithmic ASR as the dependent variable and the year of diagnosis or death as the independent variable. We found the best joinpoints (years when trends changed) using the permutation test method. Annual percentage change (APC) of each line segment between joinpoints was estimated in the model, and the APC was tested to see whether it was significantly different from 0 (*P* < 0.05). We set three joinpoints as a maximum number in each analysis. We used Stata version 12 for all analyses except the joinpoint regression analysis.^11^

## RESULTS

The characteristics of patients before and after multiple imputation are shown in Table 1. The proportion of patients with ADC increased while that with SQC and SMC decreased from the 1990s, and ADC has become a major histological type for both genders. The proportion of patients in the older age group (>75 years old) increased, while that of the younger age group (<65 years old) decreased.

**Table 1.** Characteristics of patients stratified by sex, diagnostic period, histological type, stage, and age group

	Year															

	1975–79		1980–84		1985–89		1990–94		1995–99		2000–04		2005–08		Total (1975–2008)	
							
	*n*	%	*n*	%	*n*	%	*n*	%	*n*	%	*n*	%	*n*	%	*n*	%
Males																
Histological type (before imputation^a^)																
Squamous cell carcinoma	1038	18.6	1953	25.3	2664	25.8	2997	23.9	3936	25.1	4487	23.8	3853	22.0	20 928	23.7
Adenocarcinoma	913	16.3	1702	22.0	2556	24.8	3023	24.1	4645	29.6	5909	31.3	5859	33.5	24 607	27.9
Small cell carcinoma	321	5.7	754	9.8	1124	10.9	1395	11.1	1794	11.4	2134	11.3	1843	10.5	9365	10.6
Others	221	4.0	480	6.2	700	6.8	669	5.3	780	5.0	1028	5.4	1333	7.6	5211	5.9
Missing	3095	55.4	2841	36.8	3278	31.8	4440	35.5	4530	28.9	5325	28.2	4614	26.4	28 123	31.9
Histological type (after imputation)																
Squamous cell carcinoma	2370	42.4	3200	41.4	3956	38.3	4740	37.9	5572	35.5	6423	34.0	5308	30.3	31 568	35.8
Adenocarcinoma	1954	35.0	2628	34.0	3706	35.9	4555	36.4	6432	41.0	7986	42.3	7801	44.6	35 062	39.7
Small cell carcinoma	772	13.8	1168	15.1	168	16.3	2177	17.4	2584	16.5	3072	16.3	2613	14.9	14 069	15.9
Others	492	8.8	735	9.5	977	9.5	1052	8.4	1098	7.0	1403	7.4	1780	10.2	7536	8.5
Stage^b^ (before imputation)																
Localised	657	11.8	867	11.2	1158	11.2	1340	10.7	2022	12.9	2694	14.3	2797	16.0	11 535	13.1
Regional	1525	27.3	2078	26.9	2900	28.1	3377	27.0	4209	26.8	4694	24.9	4077	23.3	22 860	25.9
Distant	1121	20.1	1949	25.2	2757	26.7	2997	23.9	3840	24.5	4929	26.1	5334	30.5	22 927	26.0
Missing	2285	40.9	2836	36.7	3507	34.0	4810	38.4	5614	35.8	6566	34.8	5294	30.3	30 912	35.0
Stage (after imputation)																
Localised	1084	19.4	1404	18.2	1756	17.0	2142	17.1	2943	18.8	3814	20.2	3497	20.0	16 640	18.9
Regional	2492	44.6	3290	42.6	4351	42.2	5380	43.0	6521	41.6	7414	39.3	6048	34.6	35 496	40.2
Distant	2012	36.0	3037	39.3	4214	40.8	5003	40.0	6220	39.7	7655	40.5	7957	45.5	36 098	40.9
Age group, years																
15–34	42	0.8	45	0.6	34	0.3	43	0.3	43	0.3	57	0.3	32	0.2	296	0.3
35–64	2077	37.2	2561	33.1	3547	34.4	4105	32.8	4684	29.9	5129	27.2	4200	24.0	26 303	29.8
65–74	2276	40.7	3040	39.3	3466	33.6	4141	33.1	5731	36.5	6853	36.3	6093	34.8	31 600	35.8
≥75	1193	21.4	2084	27.0	3275	31.7	4235	33.8	5227	33.3	6844	36.2	7177	41.0	30 035	34.0
Females																
Histological type (before imputation)																
Squamous cell carcinoma	170	8.1	285	9.7	472	11.6	504	9.9	677	10.4	759	9.4	641	8.5	3508	9.7
Adenocarcinoma	507	24.1	970	33.1	1443	35.5	1862	36.5	2850	43.8	3690	45.8	3722	49.5	15 044	41.5
Small cell carcinoma	68	3.2	215	7.3	310	7.6	390	7.6	510	7.8	537	6.7	462	6.1	2492	6.9
Others	59	2.8	119	4.1	187	4.6	205	4.0	221	3.4	287	3.6	355	4.7	1433	4.0
Missing	1296	61.7	1341	45.8	1659	40.8	2145	42.0	2246	34.5	2777	34.5	2346	31.2	13 810	38.1
Histological type (after imputation)																
Squamous cell carcinoma	454	21.6	584	20.0	801	19.7	936	18.3	1058	16.3	1241	15.4	981	13.0	6056	16.7
Adenocarcinoma	1269	60.4	1748	59.7	2420	59.5	3128	61.3	4295	66.0	5472	68.0	5275	70.1	23 607	65.1
Small cell carcinoma	224	10.7	388	13.2	550	13.5	697	13.6	811	12.5	910	11.3	747	9.9	4326	11.9
Others	153	7.3	210	7.2	299	7.4	345	6.8	341	5.2	427	5.3	523	7.0	2299	6.3
Stage (before imputation)																
Localised	206	9.8	276	9.4	395	9.7	545	10.7	954	14.7	1549	19.2	1585	21.1	5510	15.2
Regional	540	25.7	662	22.6	951	23.4	1107	21.7	1504	23.1	1493	18.6	1338	17.8	7595	20.9
Distant	426	20.3	781	26.7	1099	27.0	1251	24.5	1527	23.5	2036	25.3	2067	27.5	9187	25.3
Missing	928	44.2	1211	41.3	1626	39.9	2203	43.2	2519	38.7	2972	36.9	2536	33.7	13 995	38.6
Stage (after imputation)																
Localised	367	17.5	489	16.7	659	16.2	891	17.5	1354	20.8	2058	25.6	1914	25.4	7731	21.3
Regional	923	44.0	1169	39.9	1628	40.0	2013	39.4	2521	38.8	2716	33.7	2269	30.2	13 239	36.5
Distant	810	38.6	1272	43.4	1784	43.8	2202	43.1	2629	40.4	3276	40.7	3343	44.4	15 317	42.2
Age group, years																
15–34	32	1.5	24	0.8	25	0.6	27	0.5	27	0.4	33	0.4	25	0.3	193	0.5
35–64	798	38.0	960	32.8	1192	29.3	1460	28.6	1805	27.8	2128	26.4	1807	24.0	10 150	28.0
65–74	786	37.4	1043	35.6	1343	33.0	1511	29.6	1830	28.1	2245	27.9	2166	28.8	10 924	30.1
≥75	484	23.1	903	30.8	1511	37.1	2108	41.3	2842	43.7	3644	45.3	3528	46.9	15 020	41.4

^a^The number and proportion by stage and histological type are calculated before and after multiple imputation.

^b^Stage at diagnosis is classified into three groups: localised (cancer is confined to the organ from which is originated, regional (cancer metastasizes into regional lymph nodes and/or invades adjacent organs), distant (cancer metastasizes to distant organs and/or lymph nodes).

**Figure 1.**
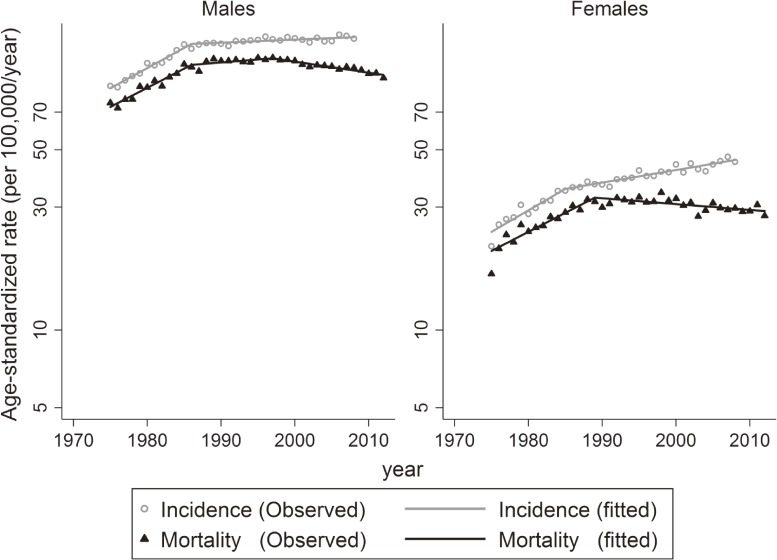
Trends in age-standardized incidence and mortality rates for lung cancer in Osaka, Japan from 1975 to 2008.

Trends in lung cancer incidence and mortality rates for all histological types are shown in Figure 1 and Table 2. Incidence rates steeply increased by 3.5% (95% CI, 2.9%–4.1%) per year for males and 3.7% (95% CI, 2.6%–4.8%) per year for females until 1985–86. Trends in incidence rates then slightly increased, as APC was 0.3% (95% CI, 0.1%–0.4%) for males and 1.1% (95% CI, 0.9%–1.3%) for females. Mortality rates levelled off from 1988 and slightly decreased from 1997 for males (APC −0.9%; 95% CI, −1.2% to −0.7%). For females, mortality rates decreased from 1989 (APC −0.5%; 95% CI, −0.8% to −0.3%).

**Table 2.** Trends in age-standardized incidence and mortality rates of lung cancer with joinpoint analysis in Osaka, Japan

	Trend 1			Trend 2			Trend 3		
		
	Years	APC	(95% CI)	Years	APC	(95% CI)	Years	APC	(95% CI)
Incidence									
Males	1975–1986	3.5^a^	(2.9, 4.1)	1986–2008	0.3^a^	(0.1, 0.4)			
Females	1975–1985	3.7^a^	(2.6, 4.8)	1985–2008	1.1^a^	(0.9, 1.3)			

Mortality									
Males	1975–1988	3.0^a^	(2.5, 3.6)	1988–1997	0.2	(−0.5, 1.0)	1997–2012	−0.9^a^	(−1.2, −0.7)
Females	1975–1989	3.5^a^	(2.6, 4.4)	1989–2012	−0.5^a^	(−0.8, −0.3)			

APC, annual percentage change; CI, confidence interval.

^a^APC is statistically significantly different from zero (*P* < 0.05).

**Figure 2.**
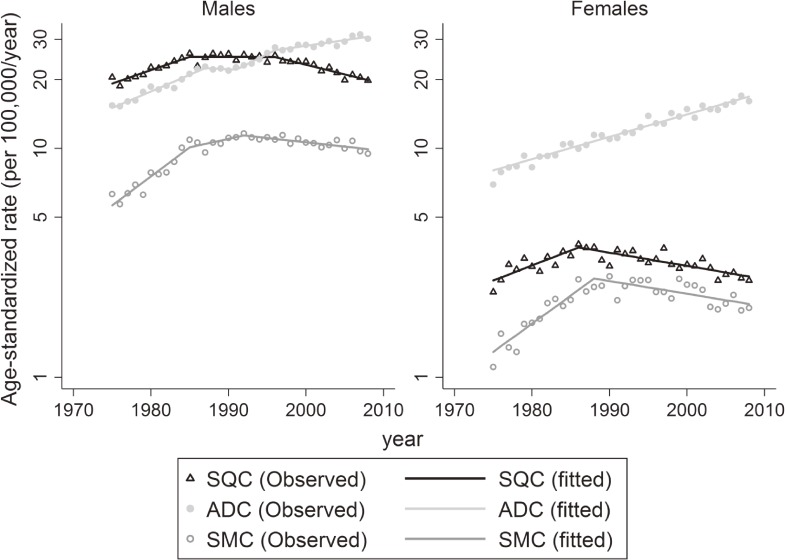
Trends in age-standardized incidence rates of lung cancer by histological type in Osaka, Japan 1975 to 2008. ADC, adenocarcinoma; SMC, small cell carcinoma; SQC, squamous cell carcinoma.

Figure 2 and Table 3 show trends in lung cancer incidence rates by histological type. The peak incidence of SQC was observed in 1996 for males and in 1986 for females. Incidence rates of SQC decreased for males (APC −1.9%; 95% CI, −2.4% to −1.5%) and females (APC −1.3%; 95% CI, −1.7% to −0.9%). ADC increased by 1.1% (95% CI, 0.8%–1.5%) for males and by 2.3% (95% CI, 2.1%–2.5%) for females. The rates of SMC decreased from 1992 for males (APC −0.9%; 95% CI, −1.3% to −0.4%) and from 1988 for females (APC −1.3%; 95% CI, −1.9% to −0.7%). The incidence rate of ADC overtook that of SQC for males in the 1990s.

**Table 3.** Trends in age-standardized incidence rates of lung cancer by histological type with joinpoint analysis in Osaka, Japan

Histological type	Trend 1			Trend 2			Trend 3			Trend 4		
			
	Years	APC	(95% CI)	Years	APC	(95% CI)	Years	APC	(95% CI)	Years	APC	(95% CI)
Males												
Squamous cell carcinoma	1975–1985	2.7^a^	(1.7, 3.7)	1985–1996	0	(−0.8, 0.7)	1996–2008	−1.9^a^	(−2.4, −1.5)			
Adenocarcinoma	1975–1987	3.4^a^	(2.7, 4.1)	1987–1991	−0.4	(−4.8, 4.3)	1991–1996	4.0^a^	(1.4, 6.6)	1996–2008	1.1^a^	(0.8, 1.5)
Small cell carcinoma	1975–1985	6.0^a^	(4.5, 7.6)	1985–1992	1.7	(−0.6, 4.1)	1992–2008	−0.9^a^	(−1.3, −0.4)			
Females												
Squamous cell carcinoma	1975–1986	3.1^a^	(1.4, 4.8)	1986–2008	−1.3^a^	(−1.7, −0.9)						
Adenocarcinoma	1975–2008	2.3^a^	(2.1, 2.5)									
Small cell carcinoma	1975–1988	5.8^a^	(3.9, 7.8)	1988–2008	−1.3^a^	(−1.9, −0.7)						

APC, annual percentage change; CI, confidence interval.

^a^APC is statistically significantly different from zero (*P* < 0.05).

We also analyzed truncated age-standardized incidence rates by histological type and age group (eFigure 1, eFigure 2, eFigure 3, eTable 1, eTable 2, and eTable 3). eFigure 1 and eTable 1 show trends in truncated (35–64 years, 65–74 years, and ≥75 years) age-standardized incidence rate of SQC by age group. For the age group 35–64 years, the rate continued to decrease in males (APC −1.0%; 95% CI, −1.3% to −0.8%), while it remained stable in females. For the age groups 65–74 years and ≥75 years, the rate significantly decreased for both genders. eFigure 2 and eTable 2 show trends in ADC. Among all age groups in males and females, except the age group 65–74 years in males, the rate significantly increased. The rate among the age group 65–74 years in males levelled off in 1997–2008. eFigure 3 and eTable 3 show trends in SMC by age group. Among the male age groups 35–64 years and 65–74 years and female age groups 65–74 years and ≥75 years, the rate decreased recently. However, the rate levelled off among the male age group ≥75 years and female age group 35–64 years.

## DISCUSSION

Although trends in incidence and mortality rates steeply increased in parallel from 1975 to the 1980s, the incidence rate increased slightly and the mortality rate decreased slightly from 1980s onwards. The latest APC of incidence rates was higher for females than males (0.3% vs 1.1%). Histologically specific analysis showed a continuous increase in ADC and a decrease in SQC and SMC since around 1990.

### Trends in the incidence and mortality rates for all histological types

Changes in incidence and mortality rates in the 1980s may be due to the decline in smoking prevalence because smoking habits are closely related to the incidence and mortality of lung cancer and other comorbidities.^3^ The increasingly widespread use of computed tomography (CT) might in part have contributed to the slight increase in incidence and the slight decrease in mortality observed from the late 1980s. It has been reported that the detection rate of CT scanning is higher than that of x-ray or sputum cytology, and cases detected by CT are likely to be early stage and peripherally located ADC.^16^^,^^17^ These cancers can be easily treated by surgery, which might have led to the decrease in mortality. However, low-dose CT screening, which was introduced in the 1990s in Japan, is experimentally conducted only in some specific areas, while CT scanning has been widely used in various clinical scenarios, such as chest pain, hemosputum, fever, faint abnormal shadow in chest x-ray, and screening for metastasis from other organ cancers. Therefore, it is difficult to evaluate the effects of CT scanning on the trends in lung cancer. The introduction and diffusion of tyrosine kinase inhibitors, such as gefitinib, which is an effective drug for advanced ADC with epidermal growth factor receptor (EGFR) mutations,^18^ might also have partially contributed to the decrease in mortality.

To better understand these incidence and mortality trends, we also confirmed trends in the proportion of early stage cancer diagnosed as a localized cancer. In population-based cancer registries in Japan, stage at diagnosis is classified into three categories: localized, regional metastases (regional lymph nodes and adjacent organs), and distant metastasis. The proportion of patients with localized cancer slightly increased from the 1990s, especially in females. Moreover, the proportion of patients with ADC was higher in females than males (Table 1). The gender differences in the distribution of histological type and stage of lung cancer might reflect different trends in incidence and mortality between genders.

### Cigarette smoking and trends in the incidence rates of SQC and SMC

It is known that the incidence of SQC and SMC is more closely related to smoking behaviors than that of ADC.^19^^,^^20^ Japanese smoking prevalence decreased from the 1960s for both genders (from 82.3% in 1965 to 38.9% in 2009 for males, and from 15.7% in 1965 to 11.9% in 2009 for females) (eFigure 4).^21^ The continuous decrease in the incidence of SQC and SMC is thought to be due to the decline in smoking prevalence.

Although the incidence rates of SQC and SMC decreased for both genders, the rates among females in the younger age group (35–64 years) levelled off (eFigure 1 and eFigure 3). Smoking prevalence among females in their 20s and 30s increased from 1965 and almost levelled off at about 17–23% in 1990–2008.^21^ It was reported that the prevalence of ever smoking by birth cohort among Japanese females continuously increased from the 1930s birth cohort and exceeded 20% by the 1973 birth cohort. Moreover, it was also reported that the mean age of smoking initiation among females declined noticeably during this period.^22^ The high smoking prevalence among females who were born after the 1960s is possibly related to the stable trends of SQC and SMC among females in the younger age group (35–64 years). The above findings suggest that it is necessary to monitor the incidence rates of SQC and SMC and to reduce smoking prevalence, especially in younger females.

Smoking prevalence for males in Osaka is almost the same as for Japan as a whole (Osaka: 48.1% in 2001 and 33.6% in 2010; Japan as a whole: 48.4% in 2001 and 33.1% in 2010), while it is a little higher among females in Osaka (Osaka: 15.7% in 2001 and 12.3% in 2010; Japan as a whole: 14.0% in 2001 and 10.4% in 2010).^23^ It is possible that the impact of smoking on lung cancer incidence is greater for females in Osaka.

### Trends in the incidence rates of ADC

The incidence rate of ADC showed an increasing trend among all the age groups for both genders except for males in the 65–74 years age group (eFigure 2). An increase in incidence of ADC has been reported worldwide, especially for females in developed countries. However, the determining factor for the increase in ADC remains unclear.^24^ It has been suggested the switching from non-filtered cigarettes to filtered cigarettes in the 1960s is related to the increase in ADC and decrease in SQC and SMC.^25^ However, smoking prevalence has decreased since the 1960s, except among younger females, and cigarette consumption per capita has levelled off and then decreased since the late 1970s (eFigure 4).^21^^,^^26^ Therefore, it is difficult to explain the increase in ADC incidence by smoking trends alone. One study has estimated the latency period between exposures to filter cigarettes and ADC development to be about 25 years,^25^ while another study suggested that it could be more than 30 years if cigarette consumption played a major role in development of ADC.^27^ If the latency period was about 30 years, the incidence rate of ADC in Osaka would be expected to have begun to level off or decrease from the 2000s.

On the other hand, ADC is the most common type of lung cancer in lifelong non-smokers.^28^^,^^29^ Specific gene mutations, such as EGFR mutations, might be related to the relationship between ADC and never smokers. It has been reported that EGFR mutations are more frequent in females, patients with ADC, never smokers, and people of East Asian ethnicity.^30^ The higher percentage of never smokers among females than males is probably one reason why the incidence of ADC increased more steeply for females than males. Passive smoking is also considered to be a risk factor for lung cancer,^31^ and an association has been clearly identified for ADC incidence (HR 2.03; 95% CI, 1.07–3.86).^32^ It is necessary to monitor trends in ADC and investigate the association between smoking and ADC incidence, with consideration of various factors, such as different distributions of EGFR mutations between ethnicities, smoking patterns, and genders.

Another possible risk factor for lung cancer is air pollution.^33^ Long-term exposure to NOx has been reported as a possible cause for a temporal increase in ADC incidence in the United States.^34^ According to a survey by the Japanese government, annual average concentrations of SO_2_ and SPM have decreased since the 1970s, while NO_2_ concentration has levelled off and slightly decreased from the 2000s (eFigure 4).^35^ Therefore, air pollution is not likely to be a major reason for the increased incidence rates of ADC.

### Comparison with the results of other studies

Our study confirmed the increase in incidence of ADC and the decrease in incidence of SQC and SMC in Osaka, which has already been shown by a previous study.^6^ According to a study of nine population-based prefectural cancer registries in Japan,^25^ incidence rates of SQC have recently decreased for both genders, which is consistent with our results in Osaka. However, the incidence of ADC in males has levelled off (APC 0.2%; 95% CI, −1.6% to 1.9%) since 1998, which is different from the results in Osaka. It seems that there are some differences in trends for lung cancer incidence between Osaka and other prefectures, especially for ADC.

According to lung cancer trends by histological type in other countries,^36^ incidence rates of SQC and SMC decreased for males and incidence of ADC increased for females in North America, Australia, and several European countries. Nevertheless, there were some differences. In North America, Australia, Denmark, and Iceland, incidence rates of ADC for males and lung cancer for all histological types for females levelled off, while in the other European countries, as in Osaka, incidence of ADC in males and lung cancer of all histological types in females increased. This is probably because smoking prevalence for both genders in North America, Australia, Denmark, and Iceland was lower and declined more sharply between 1980 and 2012 than in other countries, including Japan.^37^^,^^38^ Regarding trends in Hong Kong and Tianjin, China,^39^^,^^40^ incidence rates of lung cancer significantly decreased for both genders after the 1980s and 1990s, respectively. In these two regions, ADC incidence levelled off or decreased and SQC incidence has decreased recently for both genders. However, male smoking prevalence in China among adults aged 15 or above was high and has decreased slowly (61% in 1984 and 52.9% in 2010).^41^^,^^42^ Although smoking is closely associated with lung cancer incidence, it appears that we cannot fully understand trends in lung cancer by trends in smoking prevalence alone. Interpreting these trends more accurately requires that we also study the influence of the spread of cancer screening or exposure to other risk factors on lung cancer incidence.

### Limitations

There are several limitations to the present study. The OCR included cases with unspecified histological diagnosis. Although the percentage of cases without histological diagnosis decreased over the years, about one-third of the cases had an unspecified histological diagnosis in 2005–2008 (Table 1). We used the MI approach to solve this problem. However, when we use the MI approach, the mechanism of missingness should be missing at random, where the chance of data being missing is independent of unseen values.^10^^,^^43^ Although we assumed that this was the case for the data from OCR, it is difficult to test whether or not this assumption is valid.

The quality of cancer registry data is usually determined using the proportion of death certificate only (DCO) cases and microscopic verified (MV) cases (DCO% and MV%).^8^ For the OCR, DCO% and MV% of all cancers are as follows^44^: DCO% in 1987, 1997, and 2007 were 24%, 15%, and 11.6% for males and 21.1%, 14%, and 11.8% for females, respectively; MV% in 1992, 1997, and 2007 were 70%, 70%, and 77.6% for males and 73%, 72%, and 75.8% for females, respectively. DCO% of the OCR has improved over these periods, which might have influenced trends in incidence rates of lung cancer. It was difficult to evaluate the influence of these factors in our analysis.

### Conclusion

In this study, we investigated trends in incidence and mortality rates of lung cancer and incidence rates by histological type and age group. The incidence rates of SQC and SMC decreased with the decline in smoking prevalence, which probably led to the change in trends in lung cancer incidence rates from the mid-1980s. It was difficult to explain why the incidence rates of ADC continued to increase for both males and females. Therefore, subsequent studies should carefully monitor trends in lung cancer incidence by histological type, especially trends for ADC. The relationship between the incidence of ADC and its possible risk factors also warrants clarification.

## ONLINE ONLY MATERIALS

eTable 1. Trends in truncated age-standardized incidence rates for squamous cell carcinoma with joinpoint analysis.

eTable 2. Trends in truncated age-standardized incidence rates for adenocarcinoma with joinpoint analysis.

eTable 3. Trends in truncated age-standardized incidence rates for small cell carcinoma with joinpoint analysis.

eFigure 1. Trends in truncated age-standardized incidence rates of squamous cell carcinoma in Osaka, Japan from 1975 to 2008.

eFigure 2. Trends in truncated age-standardized incidence rates of adenocarcinoma in Osaka, Japan from 1975 to 2008.

eFigure 3. Trends in truncated age-standardized incidence rates of small cell carcinoma in Osaka, Japan from 1975 to 2008.

eFigure 4. Trends in age-standardized incidence rates of lung cancer by histological type in Osaka from 1975 to 2008, with trends in smoking prevalence and average concentration of SO_2_, NO_2_, and SPM.

Abstract in Japanese.
