# Supplementary material for: Trends in Lung Cancer Incidence Rates by Histological Type in 1975–2008: A Population-Based Study in Osaka, Japan
Source: J Epidemiol. 2016 Nov 5;26(11):579–86. doi: 10.2188/jea.JE20150257 (PMC5083321; doi:10.2188/jea.JE20150257)
Supplement: eTable 1. [file je-26-579-s001.pdf]

eTable 1. Trends in truncated age-standardized incidence rates for squamous cell carcinoma with joinpoint analysis

Age group, years	Trend 1			Trend 2			Trend 3		
	Years	APC	(95% CI)	Years	APC	(95% CI)	Years	APC	(95% CI)
**Males**									
35-64	1975-2008	-1.0 [a]	(-1.3, -0.8)						
65-74	1975-1996	0.2	(-0.4, 0.8)	1996-2008	-3.1 [a]	(-4.1, -2.0)			
≥75	1975-1984	5.9 [a]	(3.9, 7.9)	1984-1992	2.5 [a]	(0.6, 4.4)	1992-2008	-1.3 [a]	(-1.7, -0.9)
**Females**									
35-64	1975-2008	-0.5	(-1.1, 0.2)						
65-74	1975-1986	2.1	(-0.5, 4.7)	1986-2008	-1.9 [a]	(-2.6, -1.2)			
≥75	1975-1989	4.1 [a]	(2.8, 5.4)	1989-2002	-0.8	(-1.7, 0.2)	2002-2008	-5.3 [a]	(-7.6, -3.0)

APC, annual percentage change; CI, confidence interval.

[a] APC is statistically significantly different from zero ($p<0.05$)
