# Supplementary material for: Trends in Lung Cancer Incidence Rates by Histological Type in 1975–2008: A Population-Based Study in Osaka, Japan
Source: J Epidemiol. 2016 Nov 5;26(11):579–86. doi: 10.2188/jea.JE20150257 (PMC5083321; doi:10.2188/jea.JE20150257)
Supplement: eTable 2. [file je-26-579-s002.pdf]

eTable 2. Trends in truncated age-standardized incidence rates for adenocarcinoma with joinpoint analysis

Age group, years	Trend 1			Trend 2			Trend 3		
	Years	APC	(95% CI)	Years	APC	(95% CI)	Years	APC	(95% CI)
**Males**									
35-64	1975-2008	2.1 [a]	(1.9, 2.3)						
65-74	1975-1979	7.7 [a]	(2.3, 13.3)	1979-1997	1.5 [a]	(1.1, 1.9)	1997-2008	0.5	(0, 1.1)
≥75	1975-1986	6.3 [a]	(4.2, 8.5)	1986-2008	2.0 [a]	(1.6, 2.3)			
**Females**									
35-64	1975-2008	2.6 [a]	(2.3, 2.9)						
65-74	1975-2008	1.8 [a]	(1.5, 2.0)						
≥75	1975-1984	8.8 [a]	(5.2, 12.5)	1984-2008	1.8 [a]	(1.5, 2.2)			

APC, annual percentage change; CI, confidence interval.

[a] APC is statistically significantly different from zero ($p<0.05$)
