# Supplementary material for: Trends in Lung Cancer Incidence Rates by Histological Type in 1975–2008: A Population-Based Study in Osaka, Japan
Source: J Epidemiol. 2016 Nov 5;26(11):579–86. doi: 10.2188/jea.JE20150257 (PMC5083321; doi:10.2188/jea.JE20150257)
Supplement: eTable 3. [file je-26-579-s003.pdf]

eTable 3. Trends in truncated age-standardised incidence rates for small cell carcinoma with joinpoint analysis

Age group, years	Trend 1			Trend 2			Trend 3		
	Years	APC	(95% CI)	Years	APC	(95% CI)	Years	APC	(95% CI)
**Males**									
35-64	1975-1987	3.1 [a]	(1.4, 4.8)	1987-2008	-0.6 [a]	(-1.1, -0.1)			
65-74	1975-1984	6.5 [a]	(4.1, 9.0)	1984-1992	1.7	(-0.9, 4.4)	1992-2008	-1.6 [a]	(-2.1, -1.0)
≥75	1975-1990	6.3 [a]	(4.8, 7.7)	1990-2008	-0.2	(-0.7, 0.3)			
**Females**									
35-64	1975-2008	0	(-0.6, 0.7)						
65-74	1975-1983	11.2 [a]	(4.2, 18.8)	1983-2008	-1.6 [a]	(-2.4, -0.8)			
≥75	1975-1990	7.3 [a]	(4.8, 9.9)	1990-2008	-1.2 [a]	(-2.1, -0.3)			

APC, annual percentage change; CI, confidence interval.

[a] APC is statistically significantly different from zero ($p<0.05$)
