# Supplementary material for: Trends in Lung Cancer Incidence Rates by Histological Type in 1975–2008: A Population-Based Study in Osaka, Japan
Source: J Epidemiol. 2016 Nov 5;26(11):579–86. doi: 10.2188/jea.JE20150257 (PMC5083321; doi:10.2188/jea.JE20150257)
Supplement: eFigure 3. [file je-26-579-s006.pdf]

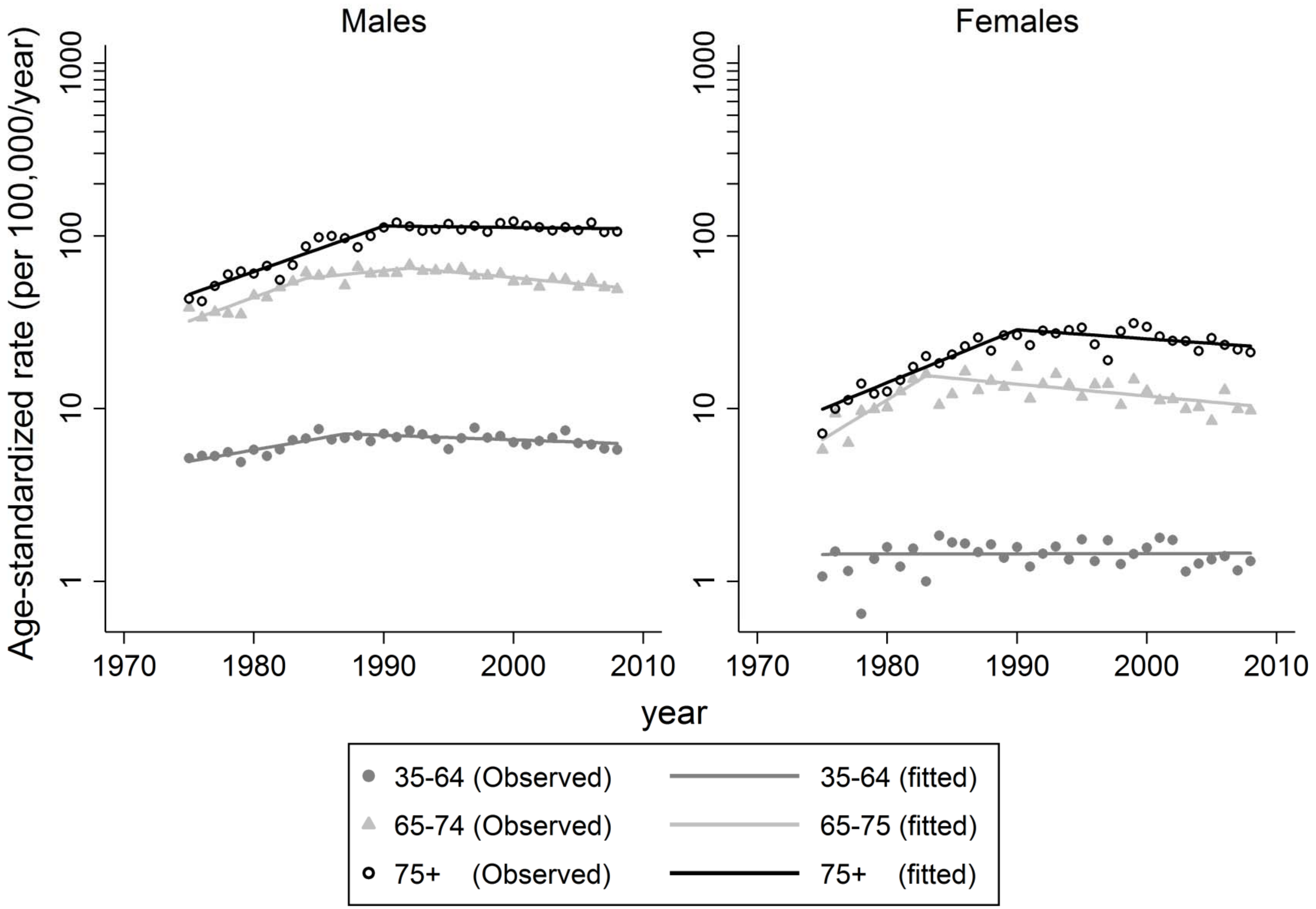

eFigure 3. Trends in truncated age-standardized incidence rates of small cell carcinoma in Osaka, Japan from 1975 to 2008
