# Supplementary material for: Trends in Lung Cancer Incidence Rates by Histological Type in 1975–2008: A Population-Based Study in Osaka, Japan
Source: J Epidemiol. 2016 Nov 5;26(11):579–86. doi: 10.2188/jea.JE20150257 (PMC5083321; doi:10.2188/jea.JE20150257)
Supplement: eFigure 4. [file je-26-579-s007.pdf]

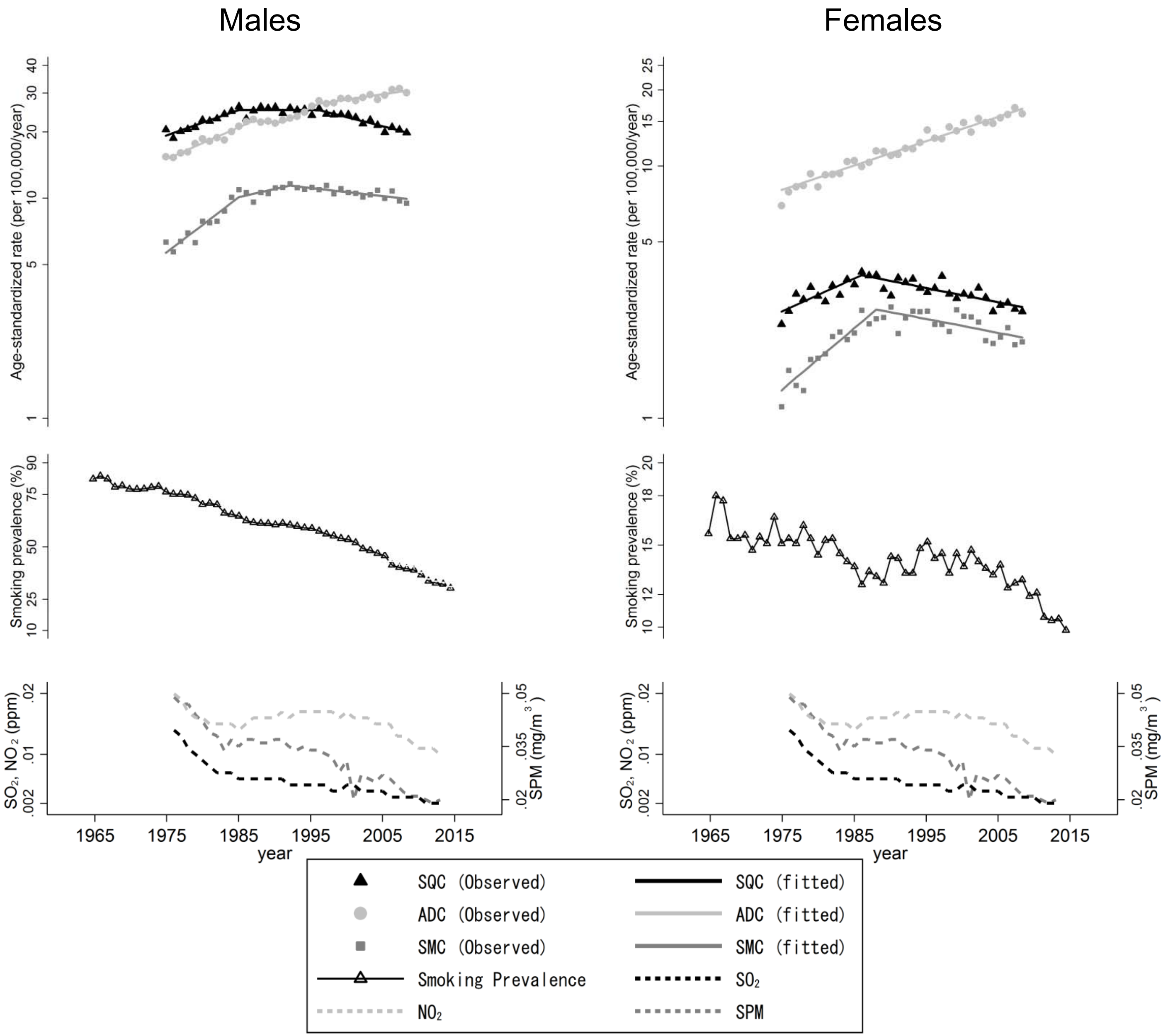


**eFigure 4.** Trends in age-standardized incidence rates of lung cancer by histological type in Osaka from 1975 to 2008, with trends in smoking prevalence and average concentration of $SO_2$, $NO_2$, and SPM

ADC, adenocarcinoma; $NO_2$, nitrogen dioxide; SMC, small cell carcinoma; $SO_2$, sulfur dioxide; SPM, suspended particulate matter; SQC, squamous cell carcinoma.
Annual trends in smoking prevalence in Japan from 1965 to 2014 were obtained from Japan smoking rate survey, Japanese Tobacco Inc, Ministry of Health, Labour and Welfare.[21]
Annual trends in average concentration of $SO_2$, $NO_2$ and SPM in Japan from 1976 to 2013 were obtained from the Environmental Statistics 2014, Ministry of the Environment.[35]
