# Supplementary material for: Trends in Lung Cancer Incidence Rates by Histological Type in 1975–2008: A Population-Based Study in Osaka, Japan
Source: J Epidemiol. 2016 Nov 5;26(11):579–86. doi: 10.2188/jea.JE20150257 (PMC5083321; doi:10.2188/jea.JE20150257)
Supplement: Abstract in Japanese. [file je-26-579-s008.pdf]

大阪府がん登録資料を用いた1975年から2008年における肺がん組織型別罹患率の推移

木下（李）福章[1]、伊藤ゆり[2]、中山富雄[2]

[1]大阪大学医学部医学科
[2]大阪府立成人病センターがん予防情報センター

背景：肺がんの罹患率や死亡率の傾向を調査することは現状のがん対策を評価していく上で重要である。本研究では、大阪府における肺がんの組織型別罹患率の推移を調査した。
方法：肺がんの罹患については大阪府がん登録より 1975～2008 年診断分のデータを入手した。肺がんの死亡については人口動態統計より 1975～2012 年死亡分のデータを入手した。joinpoint regression model を用いて、肺がん全体の年齢調整罹患率と年齢調整死亡率、また、組織型別、年齢区分別の年齢調整罹患率の推移を解析した。
結果：肺がんの年齢調整罹患率は男性においては年平均変化率 0.3% (95%信頼区間: 0.1～0.4%)で横ばい、もしくは、わずかに増加していたが、女性においては 1.1% (95%信頼区間: 0.9～1.3%)で増加していた。年齢調整死亡率は男性においては－0.9%で、女性においては－0.5%で減少していた。組織型別の解析では、扁平上皮癌と小細胞癌は男女ともに有意に減少していたが、腺癌は男女ともにほとんどすべての年齢区分で有意に増加していた。
結論：扁平上皮癌と小細胞癌の罹患率は喫煙率の減少とともに減少しており、それがおそらく、1980 年代半ば以降における肺がん全体の罹患率の傾向の変化と関係していると考えられる。しかし、腺癌が増加している原因はよくわかっていない。したがって、これからも肺がんの罹患率の傾向、特に腺癌の傾向を調査し、腺癌とその危険因子との関係を明らかにしていく必要がある。

キーワード：がん、肺がん、罹患率、組織型、がん登録
